# The Role of Metabotropic Glutamate Receptors in Social Behavior in Rodents

**DOI:** 10.3390/ijms20061412

**Published:** 2019-03-20

**Authors:** Iulia Zoicas, Johannes Kornhuber

**Affiliations:** Department of Psychiatry and Psychotherapy, University Hospital, Friedrich-Alexander-University Erlangen-Nuremberg, 91054 Erlangen, Germany; johannes.kornhuber@uk-erlangen.de

**Keywords:** metabotropic glutamate receptors, mGluR agonists, mGluR antagonists, animal models, social investigation, social recognition, aggression, anxiety

## Abstract

The appropriate display of social behavior is critical for the well-being and survival of an individual. In many psychiatric disorders, including social anxiety disorder, autism spectrum disorders, depression and schizophrenia social behavior is severely impaired. Selective targeting of metabotropic glutamate receptors (mGluRs) has emerged as a novel treatment strategy for these disorders. In this review, we describe some of the behavioral paradigms used to assess different types of social behavior, such as social interaction, social memory, aggressive behavior and sexual behavior. We then focus on the effects of pharmacological modulation of mGluR1-8 on these types of social behavior. Indeed, accumulating evidence indicates beneficial effects of selective ligands of specific mGluRs in ameliorating innate or pharmacologically-induced deficits in social interaction and social memory as well as in reducing aggression in rodents. We emphasize the importance of future studies investigating the role of selective mGluR ligands on different types of social behavior to provide a better understanding of the neural mechanisms involved which, in turn, might promote the development of selective mGluR-targeted tools for the improved treatment of psychiatric disorders associated with social deficits.

## 1. Introduction

L-glutamate represents the main excitatory neurotransmitter in the mammalian central nervous system (CNS). The actions of L-glutamate are mediated by ionotropic and metabotropic receptor subtypes (iGluR and mGluR protein families, respectively). While the ligand-gated iGluRs mediate fast synaptic responses, mGluRs slowly modulate cell excitability and synaptic neurotransmission via second messenger signaling pathways and their interaction with ion channels [1,2].

The mGluRs are members of the G-protein-coupled receptor (GPCR) superfamily and belong to class C GPCRs. These receptors are characterized by a large extracellular N-terminal domain, called the Venus flytrap domain (VFD), which contains the orthosteric ligand binding site [3]. Each VFD consists of two lobes that bind glutamate in a cleft between them. Two VFDs dimerize together and when glutamate binds to one or both VFDs large conformational changes are induced [4]. There are three main conformations of the VFD dimer: open-open, open-closed and closed-closed. The open-open conformation is the inactive conformation which is stabilized by antagonists. The open-closed conformation is induced by the binding of glutamate to one VFD, while the closed-closed conformation is induced by binding of glutamate to both VFDs. The open-closed and closed-closed conformations are active receptor conformations. In addition to glutamate, VFDs can also bind divalent cations, such as calcium and magnesium. While calcium or magnesium is required at the orthosteric ligand binding site for full receptor activation by glutamate, calcium can also bind at the allosteric binding site and activate mGluRs even in the absence of glutamate [5,6]. The allosteric ligand binding site is located topographically distinct within the transmembrane [7,8] and binds allosteric agonists, antagonists or modulators. Allosteric agonists bind to the allosteric binding site and directly activate the receptor even in the absence of an orthosteric ligand. Allosteric modulators do not activate the receptor directly but indirectly influence or modulate the effects of the orthosteric ligand. Positive allosteric modulators (PAMs), also known as allosteric enhancers or potentiators, amplify the effects of the orthosteric ligand, while negative allosteric modulators (NAMs) antagonize noncompetitively the activity of the orthosteric ligand [9]. 

The mGluRs are classified into three groups according to sequence homology, G-protein coupling and pharmacological properties. Group I includes mGluR1 and mGluR5, group II includes mGluR2 and mGluR3 and group III includes mGluR4, mGluR6, mGluR7 and mGluR8. In general, group I mGluRs couple to G_q_/G_11_ proteins and activate phospholipase C, which results in the hydrolysis of phosphoinositides and generation of diacylglycerol (DAG) and inositol 1,4,5-trisphosphate (IP_3_). DAG activates protein kinase C (PKC) and IP_3_ induces intracellular calcium release from intracellular stores and then activates PKC. Group I mGluRs can also modulate additional signaling pathways and activate a wide range of downstream effectors, including phospholipase D and several protein kinase pathways, such as casein kinase 1, Jun kinase, cyclin-dependent protein kinase 5, the mammalian target of rapamycin (mTOR)/p70 S6 kinase pathway and components of the mitogen-activated protein kinase/extracellular receptor kinase (MAPK/ERK) pathway [10,11,12]. Group II and III mGluRs are coupled predominantly to G_i/o_ proteins, which inhibit adenylyl cyclase and directly regulate ion channels and other downstream signaling molecules via release of G_βγ_ subunits. Group II and III mGluRs also activate other signaling pathways, including MAPK and phosphatidylinositol 3-kinase (PI3 kinase) pathways [13]. While group I mGluRs generally function to enhance glutamate-mediated postsynaptic excitation, group II and III mGluRs are predominantly expressed presynaptically, where they regulate neurotransmitter release [14,15]. 

The mGluRs are broadly distributed throughout the CNS and are localized at discrete synaptic and extra-synaptic sites both in neurons and glia in almost every brain region. The wide diversity and heterogeneous distribution of mGluRs provides an opportunity for selective targeting of individual mGluRs as novel treatment strategies for psychiatric and neurological disorders. Numerous studies indicate that agonists or antagonists for specific mGluR subtypes alleviate symptoms in multiple CNS disorders, including anxiety disorders [16], depression [17], schizophrenia [18,19], autism spectrum disorders [20], Alzheimer’s disease [21], Parkinson’s disease [22], fragile X syndrome [23], epilepsy [24] and pain syndromes [25]. 

The appropriate display of social behavior is critical for the survival of an organism and its reproductive success as it allows group living and successful interaction with other members of the same species, obtaining food and avoiding predation. Social behavior includes all behaviors that bring individuals together, reproductive and parental behavior, as well as all forms of aggressive behavior [26]. The importance of social behavior for the well-being of the individual is also suggested by the high number of psychiatric disorders where social deficits represent the major psychopathology, such as in social anxiety disorder [27] and autism spectrum disorders [28] or a comorbid condition, such as in depression [29], schizophrenia [29], Alzheimer’s disease [29], alcohol use disorder [30] and fragile X syndrome [31], among others.

This review will shortly describe some of the behavioral tests used to assess different types of social behavior, such as social interaction, social memory, as well as aggressive and sexual behavior. We will then focus on the effects of pharmacological modulation of different mGluR subtypes on these types of social behavior. We will also show that the discovery of selective ligands for mGluRs created potentially novel therapeutic strategies for the treatment of psychiatric disorders associated with social deficits.

## 2. Role of mGluRs in Social Interaction

### 2.1. Behavioral Tests Used to Assess Social Interaction in Rodents

Several tests have been designed to assess social interaction in rodents and they are based on the preference of rats and mice to spend time with another conspecific rather than remaining alone or on their preference to investigate social rather than non-social stimuli [32,33,34]. The most commonly used assays are the social interaction test and the three-chambered social approach test, which has several methodological variants [35]. 

The social interaction test originally designed by File and Hyde [32] consists of placing gender-matched pairs of rodents in a novel environment for 5 to 15 min and quantifying the amount of time each rodent spends interacting with or investigating his partner. The following behaviors are usually scored as indicators of social interaction: sniffing, anogenital exploration, following, social grooming, crawling under or over and playing. Possible aggressive behaviors which might occur especially between male rodents are minimized by performing the test in a novel environment. A decrease in social interaction reflects social avoidance or impaired social motivation. The social interaction test has the advantage that it mimics an ethologically natural interaction by allowing for free interaction between rodents. However, this free interaction between rodents makes it difficult to distinguish the level of social investigation that each individual initiates on its own. To allow the unbiased assessment of social investigation in each rodent separately, several tests have been designed, including the three-chambered social approach test, the social preference-avoidance test, the social approach-avoidance test, the partition test and the modified Y-maze test [35]. In these tests, the experimental rodent is able to choose to investigate or to stay away from a conspecific (social stimulus), which is enclosed in a restricted area. The social stimulus can be placed behind a transparent partition, in a wire-mesh cage or in a perforated cylinder to make sure that all social investigation is initiated by the experimental rodent. Possible aggressive or sexual interactions are prevented by enclosing the social stimulus, whereas olfactory, visual and auditory communication between the rodents remains possible [35].

The three-chambered social approach test was originally designed for mice [36,37] and later modified to work in rats [38]. The experimental apparatus consists of three chambers or compartments which can be either separated by sliding doors or remain open for the entire duration of the test. After a habituation period, an empty wire-mesh cage or a perforated cylinder is placed in one side compartment (non-social compartment), whereas a conspecific enclosed in wire-mesh cage or a perforated cylinder is placed in the other side compartment (social compartment). The experimental rodent can then explore the entire apparatus for 5 to 15 min. An increased investigation of the social stimulus (conspecific) compared with the non-social stimulus (empty cage) or an increased time spent in the social compartment compared with the non-social and center compartments reflects social preference and represents the naturally occurring behavior to be expected in most rodent species. Equal investigation of the social and non-social stimuli or equal time spent in the social and non-social compartments reflects a lack of social preference, whereas a further decline in social investigation or time spent in the social versus non-social compartment reflects social avoidance or social anxiety. Additional behaviors, such as flight, freezing, alarm cries and/or risk assessment may accompany the decreased social investigation and are stronger indicatives of social anxiety and social fear [35]. 

### 2.2. Effects of Pharmacological Modulation of Group I mGluRs on Social Interaction 

Group I mGluRs (mGluR1 and mGluR5) are distributed in several brain regions involved in social behavior and emotion regulation, such as the olfactory bulb, cortex, thalamus, hypothalamus, striatum, amygdala, hippocampus and lateral septum [39,40]. While group I mGluRs are predominantly located postsynaptically where they enhance glutamate-mediated postsynaptic excitation, mGluR1 and mGluR5 can also act presynaptically to either increase or decrease neurotransmitter release [12,14,15]. These presynaptic effects are mediated by postsynaptic group I mGluRs and release of retrograde messengers, such as endocannabinoids, or by presynaptic group I mGluRs [12,41,42]. Both mGluR1 and mGluR5 can be physically and functionally connected to ionotropic glutamate *N*-methyl-D-aspartate (NMDA) receptors and can modulate NMDA receptor function at molecular and cellular levels [43,44,45]. The mGluR5 interacts with NMDA receptors by indirect physical and positive feed-back linkage through several intracellular mechanisms, including Shank, Homer and PSD-95 proteins [46,47,48]. Activation of mGluR1 and mGluR5 leads to potentiation of NMDA currents through the activation of PKC and subsequent increase in intracellular calcium concentrations, acting thereby as indirect agonists of NMDA receptors [43,44,49,50]. This group I mGluR-mediated increase in neuronal excitability and NMDA receptor function was demonstrated in brain regions regulating social behavior, such as the hippocampus and striatum [44,49,50,51], but also in hypothalamic and cortical neurons [52,53] and hippocampal slices [54]. Altered NMDA receptor function was linked to social impairments in several models of impaired social behavior [55,56,57,58,59] and the mGluR modulation of NMDA receptors offers a potential strategy to treat these social deficits. Actually, pharmacological blockade of mGluR1 and mGluR5 function emerged as one of the most promising therapeutic strategies for the treatment of several psychiatric conditions, including disorders associated with social deficits, such as autism, schizophrenia and depression [60,61,62]. This approach revealed anxiolytic properties in several studies assessing social interaction (Table 1). For instance, the mGluR1 antagonist (3,4-dihydro-2H-pyrano[2,3-b]quinolin-7-yl)-(cis-4-methoxycyclohexyl)-methanone (JNJ16259685) reversed the deficits in social interaction in Eif4ebp2 knock-out mice [63], which show an autistic-like phenotype characterized by impaired social interaction, repetitive behaviors and vocalization defects [64,65]. In this study, JNJ16259685 increased social investigation only in autistic mice, while an identical dose in wild-type C57BL/6J mice had no effects and a higher dose even reduced social investigation [63]. However, in Shank2 knock-out rats, which also show an autistic-like phenotype, JNJ16259685 did not improve the deficits in social investigation [66]. Given that autistic Shank2 knock-out mice show decreased NMDA receptor function in the hippocampus, which could be normalized by a PAM of mGluR5 [56], and that a decreased NMDA receptor function was associated with social deficits [55,57,59], the question remains whether agonists of mGluR1 or PAMs of mGluR1 might prove more efficacious in reversing the social deficits in Shank2 knock-out rats. Another selective mGluR1 antagonist, 2-cyclopropyl-5-[1-(2-fluoro-3-pyridinyl)-5-methyl-1H-1,2,3-triazol-4-yl]-2,3-dihydro-1H-isoindol-1-one (CFMTI) improved the social interaction deficits induced by the NMDA receptor antagonist 5R,10S-(+)-5-methyl-10,11-dihydro-5H-dibenzo[a,d]cyclohepten-5,10-imine hydrogen maleate (MK-801) in rats [67], further demonstrating the potential of mGluR1 antagonists in reversing pathological social interaction. When compared to mGluR5, the mGluR1 was characterized much less in the context of emotion and behavior, which might also be due to studies reporting cognitive dysfunctions in mice lacking mGluR1 or potentially induced by mGluR1 antagonists [68,69,70]. Although several studies reported cognitive deficits following mGluR5 antagonists [71,72,73,74], they tend to emerge only following high doses and are less severe than that of mGluR1 antagonists.

The mGluR5 antagonist 3-(3-chlorophenyl)-1-(1-methyl-4-oxo-5H-imidazol-2-yl)urea (fenobam) reversed the deficits in social interaction in autistic Eif4ebp2 knock-out mice without affecting social investigation in wild-type C57BL/6J mice [63]. Interestingly, this effect was observed 24 h but not 30 min after fenobam administration and the reason for the delayed effect of fenobam is yet unclear, as another antagonist of mGluR5, 2-methyl-6-phenylethynyl-pyridine (MPEP) and a NAM of mGluR5, ((4-difluoromethoxy-3-(pyridine-2-ylethynyl)phenyl)5H-pyrrolo[3,4-b]pyridine-6(7H)-yl methanone) (GRN-529) have short-term effects on social interaction in other ASD models. As such, MPEP normalized social investigation deficits in autistic BTBR T+tf/J mice [75] and in autistic IRSp53 knock-out mice [58] and also normalized autistic-like alterations caused by prenatal exposure to the anticonvulsant drug valproic acid in rats [76]. Pretreatment with MPEP was also successful in preventing social interaction deficits induced by a 10-day methamphetamine injections and a 10-day withdrawal regimen in mice [77] and in increasing social investigation in inbred Balb/c mice, which show impaired social interaction [78], further demonstrating its therapeutic potential. Besides normalizing the deficits in social interaction, treatment of autistic IRSp53 knock-out mice, which display enhanced NMDAR function in the hippocampus, with MPEP (which indirectly inhibits NMDA receptor function; see above) normalized NMDA receptor function and plasticity in the hippocampus and neuronal firing in the medial prefrontal cortex [55,58]. Similar to the effects of MPEP, the deficits in social interaction in autistic BTBR T+tf/J mice could also be reversed by the mGluR5 NAM GRN-529 [79]. Interestingly, [3-cyano-*N*-(1,3-diphenyl-1H-pyrazol-5-yl) benzamide] (CDPPB), a PAM of mGluR5 was also able to normalize social interaction deficits in autistic Shank2 knock-out mice [56] and in autistic Sarm1 knock-out mice [80] and normalized autistic-like alterations caused by prenatal cannabinoid exposure in rats [81]. These effects of CDPPB on social behavior are not so surprising given that autistic Sarm1 knock-out mice and prenatally cannabinoid-exposed rats show a down-regulation of mGluR5 expression and an impaired mGluR-dependent long-term depression (LTD) in the hippocampus and the medial prefrontal cortex, respectively, which was normalized by enhancement of mGluR5 signaling through CDPPB [80,81]. Autistic Shank2 knock-out mice, on the other hand, show decreased NMDA receptor function in the hippocampus, which was normalized by enhancement of mGluR5 signaling through CDPPB [56]. These results, together with the altered NMDA receptor function implicated in social impairments [55,56,57,59], suggest that deviation in NMDA receptor function in either direction leads to social deficits and that correcting this deviation with group I mGluR compounds has beneficial effects [58]. Interestingly, the social interaction deficits induced by the NMDA receptor antagonist MK-801 in rats were not only reversed by the PAM of mGluR5 CDPPB, but also by a concomitant administration of sub-effective doses of CDPPB and N,N′-Dicyclopentyl-2-methylsulfanyl-5-nitro-pyrimidine-4,6-diamine (GS39783), a PAM of gamma-Aminobutyric acid (GABA) B receptor, suggesting that simultaneous activation of mGluR5 and GABAB receptors might also correct the deviation in NMDA receptor function [82]. 

### 2.3. Effects of Pharmacological Modulation of Group II mGluRs on Social Interaction 

Group II mGluRs (mGluR2 and mGluR3) are distributed in brain regions associated with social behavior and emotion regulation, such as the prefrontal cortex, anterior cingulate cortex, thalamus, amygdala and hippocampus [83,84]. While group II mGluRs are predominantly located presynaptically where they function as auto- and hetero-receptors and inhibit the release of glutamate and other neurotransmitters, mGluR2 and mGluR3 are also found in postsynaptic and glial localizations [85,86]. In fact, the selective group II mGluR agonist (–)-2-Oxa-4-aminobicyclo[3.1.0]hexane-4,6-dicarboxylic acid (LY379268) was shown to enhance postsynaptic NMDA receptor function in prefrontal [87] and hippocampal [88] neurons via activation of the Akt/GSK-3β signaling pathway and Src kinase, respectively. Differentiation between these two receptors has been quite difficult due to lack of pharmacological tools. Studies using the mGluR2/3 agonist (1S,2S,5R,6S)-2-Aminobicyclo[3.1.0]hexane-2,6-dicarboxylic acid (LY354740) in mGluR2 knock-out and mGluR3 knock-out mice showed that, although mGluR3 plays a role in anxiety-related behaviors, most of the anxiolytic effects in non-social contexts rely on mGluR2 [89,90]. Anxiolytic-like effects of LY354740 have also been reported in social contexts, where pretreatment with LY354740 prevented sodium-lactate-induced decrease in social interaction in rats [91] (Table 1). Another mGluR2/3 agonist, LY379268, reversed the deficits in social interaction induced by toluene [92] and by prenatal repeated episodes of restraint stress in mice [93]. These prenatally restrained mice also showed decreased mGluR2 and mGluR3 mRNA and protein levels in the frontal cortex, which could be normalized by LY379268 [93]. The efficacy of mGluR2/3 antagonists has also been tested in both the social interaction test and the three-chambered social approach test. For instance, [(2S)-2-amino-2-[(1S,2S)-2-carboxycycloprop-1-yl]-3-(xanth-9-yl) propanoic acid] (LY341495) improved social deficits in depressive dS-Shati/Nat8l mice which exhibited behavioral despair in the forced swimming and tail suspension tests and social withdrawal in the three-chambered social approach test [94]. Interestingly, another mGluR2/3 antagonist (1R,2R,3R,5R,6R)-2-Amino-3-(3,4-dichlorobenzyloxy)-6-fluorobicyclo[3.1.0]hexane-2,6-dicarboxylic acid (MGS0039) did not increase social interaction in rats, although MGS0039 and LY341495 show comparable affinity for mGluR2 and mGluR3 in rats [95]. Given that LY341495 also shows affinity for mGluR7 and mGluR8 [83] and that MGS0039 shows a lower affinity for mGluR7 than LY341495 [95], mGluR7 and mGluR8 might partly mediate the effects of LY341495 on social interaction. 

### 2.4. Effects of Pharmacological Modulation of Group III mGluRs on Social Interaction 

Like group II, group III mGluRs (mGluR4, mGluR6, mGluR7 and mGluR8) are predominantly expressed presynaptically where they function as auto- and hetero-receptors and regulate neurotransmitter release [14]. Except for mGluR6 whose expression is restricted to the retina, all other group III mGluRs are expressed in brain regions involved in social behavior and emotion regulation. As such, mGluR4 are highly expressed in the olfactory bulb, cortex, thalamus, hippocampus and basal ganglia [96], mGluR7 are expressed in the olfactory bulb, cortex, hippocampus, amygdala, thalamus and hypothalamus [97], whereas mGluR8 are expressed in the olfactory bulb, cortex and hippocampus [98]. Group III mGluRs received less attention than group I and group II mGluRs mostly due to shortage of selective pharmacological agents to study them [99,100]. Nevertheless, a few studies investigated the effects of group III mGluR modulation on social interaction (Table 1). For instance, chronic facilitation of mGluR4 signaling with the PAM *cis*-2-[[(3,5-Dichlorophenyl)amino]carbonyl]cyclohexanecarboxylic acid sodium salt (VU0155041) normalized the social interaction deficits in autistic Oprm1 knock-out mice lacking the mu opioid receptor gene [101]. Similarly, other studies have shown that activation of mGluR4 with the mGluR4 agonist (2S)-2-amino-4-({[4-(carboxymethoxy)phenyl](hydroxy)methyl}(hydroxy)phosphoryl)butanoic acid (LSP4-2022) and the PAMs of mGluR4 (1S,2R)-N1-(3,4-dichlorophenyl)cyclohexane-1,2-dicarboxamide (Lu AF21934) and (E)-4-(2-Phenylethenyl)-2-pyrimidinamine (Lu AF32615) reduced the social interaction deficits induced by the NMDA receptor antagonist MK-801 in rats [102,103,104]. Interestingly, the social interaction deficits induced by MK-801 were not only prevented by pretreatment with LSP4-2022 in mice, but also by a concomitant administration of sub-effective doses of LSP4-2022 and 3-Amino-*N*-(4-methoxybenzyl)-4,6-dimethylthieno[2,3-*b*]pyridine carboxamide (VU152100), a PAM of muscarinic M4 receptor, suggesting that simultaneous activation of mGluR4 and M4 receptors might be beneficial in preventing social interaction deficits elicited by an impaired NMDA receptor function [105].

The positive effects of mGluR7 and mGluR8 antagonism on social interaction were indicated by Chaki et al. [95], who showed that the mGluR2/3 antagonist LY341495, which also shows affinity for mGluR7 and mGluR8 [83], increased social interaction in rats. In accordance with this study, mGluR8 knock-out mice showed enhanced social interaction, although measures of non-social anxiety as assessed in the elevated plus maze, open field and acoustic startle response were increased [106]. Up to date, there have been only two mGluR7 NAMs synthetized—[6-(4-Methoxyphenyl)-5-methyl-3-(4-pyridinyl)-isoxazolo(4,5-c)pyridin-4(5H)-one (MMPIP) and [6-(2,4-Dimethylphenyl)-2-ethyl-6,7-dihydro-4(5*H*)-benzoxazolone] (ADX71743) [107,108]—and although the affinity of MMPIP is slightly better than that of ADX71743, both compounds are very potent at mGluR7 [109]. While ADX71743 prevented MK-801-induced social interaction deficits in mice, MMPIP did not [109], which might be due to the different pharmacokinetic profile of the compounds: ADX71743 exhibits high kinetic solubility but low metabolic stability, while MMPIP shows better metabolic stability but lower biological activity and solubility [109].

## 3. Role of mGluRs in Social Memory

### 3.1. Behavioral Tests Used to Assess Social Memory in Rodents

For most social animals, including humans and rodents, recognition of conspecifics is crucial for the development of normal social relationships and for the establishment of hierarchies that limit aggressive interactions and allow group living [113,114]. Whereas in humans and other primates individual recognition is achieved by using visual and auditory cues, in most non-primate mammals social recognition is achieved through information encoded by olfactory and pheromonal signals [115]. The assessment of social memory in rodents is based on the tendency of rats and mice to investigate unfamiliar conspecifics more intensely than familiar ones. Social memory is indicated by a decline in the amount of investigation of previously encountered conspecifics [116], which are usually juveniles or ovariectomized females to prevent possible aggressive and/or sexual behaviors. Two main test paradigms have been developed to measure social memory in rodents, namely the social recognition test and the social discrimination test.

The social recognition test can be performed in two main ways. In the first variant, which is based on a habituation-dishabituation paradigm, the experimental rodent is repeatedly exposed to the same conspecific (up to four trials), which leads to a decrease in the amount of olfactory investigation directed towards this conspecific. During the fifth trial, the experimental rodent is exposed to an unfamiliar conspecific and, if social memory is intact, investigation of the unfamiliar conspecific returns to investigation levels shown during the first trial [117]. Social investigation includes sniffing, anogenital exploration, grooming and close following of the social stimuli. In the second variant, the experimental rodent is exposed to the same conspecific twice, with a short inter-exposure interval [118,119]. Social recognition is indicated in this case by a reduced investigation of the conspecific during the second trial as compared with the first trial. However, there are a number of limitations in the use of the social recognition test, especially in its first variant. For example, repeatedly using the same conspecific as a social stimulus may lead to sensitization to the testing procedure, which in turn may mask specific individual recognition-related behavioral changes. A lack of dishabituation response during exposure to the unfamiliar conspecific may therefore not only indicate impaired social memory but also disinterest, fatigue or even social anxiety.

In the social discrimination test, the experimental rodent is exposed to an unknown conspecific for 4 to 10 min and, after a certain inter-exposure interval, is presented with this same social stimulus along with a novel stimulus for the same amount of time. The social discrimination test allows in this way to assess the ability of rodents to distinguish between two social stimuli within the same time. During the second trial, the rodent is provided with a simultaneous binary choice between the familiar (same) and the unfamiliar (novel) conspecific [120,121]. In a variant of this test, the rodent is exposed to an unknown conspecific for 30 min and a second (novel) conspecific is introduced at the end of this period for another 5 min [122]. An increased investigation of the novel conspecific compared with the same conspecific indicates intact social memory and discrimination abilities. 

### 3.2. Effects of Pharmacological Modulation of mGluRs on Social Memory

There are few studies investigating the effects of mGluRs on social memory and most of them assessed the function of group I and group II mGluRs (Table 2). As such, Clifton et al. [121] have shown that activation of mGluR5 with the PAMs CDPPB and (*S*)-(4-fluorophenyl)-3-[3-(4-fluoro-phenyl)-[1,2,4]-oxidiazol-5-yl]piperidon-1-yl)methanone (ADX47273) enhanced social discrimination and reversed social discrimination deficits induced by neonatal phencyclidine (PCP) treatment in rats. Neonatal PCP treatment was shown to induce a schizophrenia-like phenotype in rodents, which is characterized by hyperactivity, impaired social interaction and memory [123], and to directly block NMDA receptors and to reduce glutamatergic transmission [124]. Like in social interaction, mGluR5 seems to interact with NMDA receptors to modulate social memory. As such, CDPPB was shown to improve social discrimination deficits elicited by the NMDA receptor antagonist MK-801 and, reciprocally, pretreatment with MK-801 or the mGluR5 antagonist MPEP (which indirectly inhibits NMDA receptor function; see Section 2.2.) blocked the effects of CDPPB on social discrimination in rats [121].

Regarding the involvement of group II mGluRs in social memory, Harich et al. [122] have shown that activation of mGluR2/3 with the mGluR2/3 agonist LY354740 and the PAM of mGluR2 2,2,2-Trifluoro-*N*-[4-(2-methoxyphenoxy)phenyl]-*N*-(3-pyridinylmethyl)ethanesulfonamide hydrochloride (LY487379) reversed the deficits in social discrimination induced by neonatal PCP treatment in rats. Similarly, the mGluR2/3 agonist LY379268 and the mGluR2 PAMs 3′-[[(2-cyclopentyl-2,3-dihydro-6,7-dimethyl-1-oxo-1H-inden-5-yl)oxy]methyl]-[1,1′-biphenyl]-4-carboxylic acid (BINA) and (2S)-5-methyl-2-{[4-(1,1,1-trifluoro-2-methylpropan-2-yl)phenoxy]methyl}-2,3-dihydroimidazo[2,1-b][1,3]oxazole-6-carboxamide (TASP0443294) improved social recognition impairments elicited by MK-801 in rats [119,125]. Given that this improvement in social memory induced by BINA was blocked by pretreatment with the mGluR2/3 antagonist LY341495 [119], the effects of mGluR2/3 agonists on social memory impairment seem to be mediated via mGluR2. Interestingly, blockade of mGluR2/3 activity by the mGluR2/3 antagonist MGS0039 was also shown to enhance social recognition in rats probably through stimulation of the postsynaptic ionotropic glutamate receptor α-amino-3-hydroxy-5-methyl-4-isoxazolepropionic acid (AMPA), as pretreatment with the AMPA receptor antagonist 2,3-Dihydroxy-6-nitro-7-sulfamoylbenzo(f)-quinoxaline (NBQX) attenuated the effects of MGS0039 on social recognition [118]. Furthermore, while the mGluR2/3 agonists and the mGluR2 PAMs were administered before testing [119,122,125], MGS0039 was administered immediately after the first encounter with the social stimulus [118], suggesting that mGluR2/3 agonists and antagonists may differentially affect the learning and consolidation phases of the memory process, respectively. 

## 4. Role of mGluRs in Male Aggressive Behavior

### 4.1. Behavioral Tests Used to Assess Aggressive Behavior in Male Rodents

Aggressive behavior in male rodents is usually related to territorial defensive behavior or to the establishment and maintenance of social status within a group [126]. Rats and mice differ in their social organization and use of aggressive behaviors. While mice are territorial and do not tolerate unfamiliar males within their home cage, rats live in groups and tend to coexist peacefully if the composition of the group is stable. Although both mice and rats establish social dominance hierarchies within groups, rats form stable hierarchies which tend to remain the same, whereas mice establish instable hierarchies where individuals keep fighting and continuously compete for dominance [126]. Changes in group composition, as well as the presence of females or manipulation of the mice, such as cage changing or temporary removal for experimental procedures might increase fighting. These characteristics of male mice make them an attractive model to study aggressive behavior. There are two main tests used to assess aggressive behavior in male rodents, namely the standard opponent test and the resident intruder test.

In the standard opponent test two unfamiliar conspecifics are placed in a neutral arena and the frequency and latency of agonistic behavior, such as threats, attacks and bites are assessed [127]. The opponents are selected for highly replicable behavior as either submissive or dominant from mouse strains known for either low or high levels of aggression, respectively. Alternatively, two unfamiliar same-strain conspecifics which received the same treatment can be used as partners. The test often takes between 5 to 15 min but can be terminated earlier when the attacks and bites are severe. A modification of the opponent test is the isolation-induced aggression, where males are single housed for a certain period of time, usually for several weeks, before placing them with an unfamiliar male in a neutral arena [128]. This isolation was shown to increase the frequency of fighting and attacks. 

In the resident intruder test an unfamiliar male (intruder) is placed in the home cage of the experimental rodent (resident), which defends its territory and shows aggressive behavior towards the intruder [129,130]. Higher levels of aggression might be obtained when residents are co-housed with females. 

### 4.2. Effects of Pharmacological Modulation of mGluRs on Male Aggressive Behavior

Several studies indicated a role of mGluRs in the regulation of aggressive behavior in mice (Table 3). As such, inhibition of mGluR1 activity by the mGluR1 antagonist JNJ16259685 reduced isolation-induced aggression in mice [131]. A similar role in aggression regulation was attributed to mGluR5, where inhibition of mGluR5 activity by the mGluR5 antagonists MPEP and 3-((Methyl-1,3-thiazol-4-yl)ethynyl) pyridine hydrochloride (MTEP) decreased isolation-induced aggression [127] and aggressive behavior in the resident-intruder test in mice [129], respectively. Interestingly, while MPEP decreased offensive behaviors it also increased exploration and social investigation [127], supporting studies showing that MPEP increases social investigation in models of impaired social interaction [58,75,76,77,78] (Table 1). 

The mGluR2/3 and mGluR7 were attributed an anti-aggressive function, whereas mGluR8 does not seem to regulate aggressive behavior. As such, it was shown that the mGluR2/3 agonist LY379268 reduced isolation-induced aggression in mice [128] and aggressive behavior in the resident-intruder test [129]. Similarly, the mGluR7 agonist *N*,*N*′-*Bis*(diphenylmethyl)-1,2-ethanediamine dihydrochloride (AMN082) reduced aggressive behavior in the standard opponent test, but did not affect social investigation in these mice [132]. Interestingly, mGluR7 knock-out mice show a severe reduction in aggressive behavior correlated with an olfactory deficit, which is expressed as reduced attention toward male urine and reduced c-Fos expression induced by urine exposure in the bed nucleus of the stria terminalis (BNST) [130]. Given that olfaction is critical for the appropriate display of social behavior in rodents, including social recognition and initiation of aggressive behaviors, these results indicate that mGluR7-mediated olfactory processing in the BNST is essential for inter-male aggression [130]. Intra-BNST administration of the mGluR7 antagonist MMPIP in wild-type mice reproduced the phenotype of mGluR7 knock-out mice, highlighting again the importance of BNST mGluR7 for the expression of aggressive behavior [130]. Studies to date suggest that mGluR8 might not regulate aggressive behavior. As such, mGluR8 agonist (*S*)-3,4-Dicarboxyphenylglycine ((S)-3,4-DCPG) and the combined mGluR8 agonist and AMPA receptor antagonist (*RS*)-3,4-Dicarboxyphenylglycine ((RS)-3,4-DCPG) did not affect aggressive behavior or social investigation in the standard opponent test [132,133]. However, additional experiments using other selective ligands for mGluR8 are needed to confirm these results. 

## 5. Role of mGluRs in Male Sexual Behavior

As already mentioned, mGluR ligands proved to be effective in reducing pathological symptoms in several rodent models of psychiatric disorders, including anxiety disorders, depression, schizophrenia, autism spectrum disorders and fragile X syndrome [16,17,18,19,20,23]. However, given that a number of drugs used to treat these disorders have unwanted effects on natural reward, such as food and sex [134,135,136], the question arises as to whether mGluR compounds might have similar unwanted effects on male sexual behavior. Male sexual behavior involves a pattern of genital and somatomotor responses which are elicited and maintained by pheromonal, neural and hormonal signals. It includes copulation and pre-copulatory behaviors that allow males to detect a mate and stimulate a receptive response [137]. The copulatory behavior consists of mounts, intromissions and ejaculation and can be assessed by exposing males to receptive females [138]. Parameters such as mount latency and intromission latency are indicative of sexual motivation, whereas parameters such as latency to ejaculation, number of mounts and intromissions before ejaculation and post-ejaculation interval (the time from ejaculation to the next intromission) are measures of sexual performance [137]. Sex-seeking behavior can be assessed by placing a receptive female in the mating cage before the behavioral testing is started to provide female olfactory stimuli. The male is then placed in this mating cage for a certain time before the female is reintroduced and the sex-seeking behavior is assessed by counting the number of movements between two distinct parts of the cage [139]. 

Several studies to date investigated the effects of mGluR compounds on male sexual behavior (Table 4). As such, Seredynski et al. [140] showed that the mGluR1 antagonist [(S)-(+)-α-amino-4-carboxy-2-methylbenzeneacetic acid] (LY367385) inhibited sexual motivation in male Japanese quails, while the mGluR5 antagonist MPEP and the mGluR2/3 antagonist LY341495 did not affect sexual motivation [140]. Given that the mGluR1 antagonist LY367385 blocked the reversal of sexual motivation in castrated Japanese quails induced by the estrogen receptor β (ERβ)-specific agonist diarylpropionitrile indicates that ERβ interacts with mGluR1 signaling to regulate male sexual motivation [140]. This is further supported by the fact that both ERα and ERβ stimulate different group I and II mGluRs in a brain-region specific manner [141]. Other studies also investigated male sexual behavior after administration of mGluR5 antagonists and NAMs of mGluR5 in rats. As such, treatment with the mGluR5 antagonists MPEP and MTEP and the mGluR5 NAM GRN-529 did not affect sexual behavior in rats [139,142,143], whereas MPEP administration decreased sex-seeking behavior and inhibited sexual behavior at high doses only [139]. Similar to mGluR5, mGluR2/3 do not seem to regulate sexual behavior in males, as neither the mGluR2/3 antagonist LY341495 [140] nor the mGluR2/3 agonist LY379268 [139] affected sexual motivation and sexual performance in Japanese quails or sex-seeking behavior in rats, respectively. The mGluR7 agonist AMN082 decreased sex-seeking behavior and inhibited sexual behavior only at high doses [139], indicating that lower doses do not alter sexual behavior in rats. Taken together, these results suggest that, except for mGluR1 antagonists, other mGluR compounds tested to date do not induce negative effects on male sexual behavior. However, additional experiments using other selective ligands for these mGluRs are needed to confirm these results.

## 6. Concluding Remarks

Our understanding of the mGluRs and their involvement in health and disease has grown exponentially within the last years. Several lines of evidence suggest a beneficial effect of selective ligands of specific mGluRs on abnormal social behavior, such as impaired social interaction, deficits in social memory or heightened aggression. Pharmacological modulation of group I mGluRs, especially of mGluR5, represents one of the most promising strategies for the normalization of pharmacologically-induced or innate deficits in social interaction as well as for reducing aggression, either through direct mGluR-mediated actions or through indirect NMDA receptor-mediated actions. Although positive modulation of group II mGluRs was proven effective in normalizing pharmacologically-induced social deficits and heightened aggression, most studies investigated their potential in ameliorating the pharmacologically-induced deficits in social memory. Group III mGluR-selective compounds received less attention mostly due to shortage of selective pharmacological agents to study them and seem to be less involved in social behavior as compared with group II and group III mGluRs. However, future studies are needed to delineate the involvement of group III mGluRs in different types of social behavior. This emerging evidence makes further studies investigating the potentially beneficial role of selective modulation of mGluRs on social behavior worthwhile. Apart from providing a better understanding of the neural mechanisms involved and regulated by mGluRs, these investigations might stimulate the development of selective mGluR-targeted tools for the improved treatment of psychiatric disorders associated with social deficits.

## Figures and Tables

**Table 1:** Effects of pharmacological modulation of mGluRs on social interaction.

Action	Drug	Species	Behavioral Test	Dose Used	Behavioral Change	References
mGluR1 antagonist	JNJ16259685	Shank2 KO rats	three-chambered SAT and SIT	0.63 mg/kg, s.c. 30 min before test	Did not improve social impairments	[66]
		Eif4ebp2(-/-) mice	three-chambered SAT	0.3 mg/kg, i.p. 30 min before test	Increased sniffing of social stimulus and time in social compartment	[63]
		C57BL/6J mice	three-chambered SAT	0.3 and 1 mg/kg, i.p. 30 min before test	1 mg/kg but not 0.3 mg/kg reduced time in social compartment	[63]
mGluR1 antagonist	CFMTI	Sprague-Dawley rats	SIT	3 and 10 mg/kg, p.o. 30 min before test, 4 h after MK-801	Reduced MK-801-induced deficits in social interaction	[67]
mGluR5 antagonist	fenobam	Eif4ebp2(-/-) mice	three-chambered SAT	3 mg/kg, i.p. 30 min before test	Increased sniffing of social stimulus and time in social compartment	[63]
		C57BL/6J mice	three-chambered SAT	3 and 10 mg/kg, i.p. 30 min before test	10 mg/kg but not 3 mg/kg reduced time in social compartment	[63]
mGluR5 antagonist	MPEP	BTBR T+tf/J mice	three-chambered SAT	1, 10 and 30 mg/kg, i.p. 30 min before test	Only 30 mg/kg increased sniffing of social stimulus, but did not change time in social compartment	[75]
		IRSp53(-/-) mice	three-chambered SAT	10 and 30 mg/kg, i.p. 10 min before test	30 mg/kg but not 10 mg/kg increased sniffing of social stimulus and time in social compartment	[58]
		Prenatally VPA-treated rats	three-chambered SAT	30 mg/kg, i.p. 30 min before test	Increased social preference	[76]
		Balb/c mice	three-chambered SAT	30 mg/kg, i.p. 20 min before test	Increased sniffing of social stimulus, but unchanged time in social compartment	[78]
		Swiss Webster mice	three-chambered SAT	30 mg/kg, i.p. 20 min before test	Decreased sniffing of social stimulus, but did not change time in social compartment	[78]
		Lister Hooded rats	SIT	0.3, 1 and 10 mg/kg, p.o. 1 h before test	0.3 and 1 mg/kg but not 10 mg/kg increased social investigation	[110]
mGluR5 antagonist	MTEP	Wistar rats	SIT	1, 3 and 10 mg/kg, i.p. 60 min before test	3 and 10 mg/kg but not 1 mg/kg decreased social interaction	[111]
mGluR5 PAM	CDPPB	Wistar rats	SIT	0.75 mg/kg, i.p. 30 min before test	Normalized social interaction deficits induced by prenatal cannabinoid exposure	[81]
		Wistar rats	SIT	0.25, 0.5 and 1 mg/kg before test, 3.5 h after MK-801	Reduced MK-801-induced deficits in social interaction	[82]
		Sarm1 KO mice	SIT	10 mg/kg, i.p 6 h before test	Decreased freezing during social interaction	[80]
		Shank2 KO mice	SIT and three-chambered SAT	3 and 10 mg/kg, i.p. 30 min before test	10 mg/kg but not 3 mg/kg increased sniffing of social stimulus	[56]
mGluR5 NAM	GRN-529	BTBR T+tf/J mice	three-chambered SAT	0.3, 1 and 3 mg/kg, i.p. 30 min before test	Increased sniffing of social stimulus (all doses) and time in social compartment (only 3 mg/kg)	[79]
mGluR2/3 agonist	LY354740	Sprague-Dawley rats	SIT	0.3 and 0.6 mg/kg, i.p. 1 h before lactate	Decreased (0.3 mg/kg) and blocked (0.6 mg/kg) lactate-induced decrease in social interaction	[91]
mGluR2/3 agonist	LY379268	NMRI mice	SIT	250, 500 and 750 mg/kg, i.p. 30 min before toluene, 30 min before test	Only 750 mg/kg normalized toluene-induced deficits in social interaction	[92]
		Mice	SIT	0.5 mg/kg, i.p. for 5 days, twice per day	Increased social interaction in unstressed mice and in mice prenatally exposed to repeated episodes of restraint stress	[93]
mGluR2/3 antagonist	LY341495	dS-Shati/Nat8l mice	three-chambered SAT	0.3 mg/kg, i.p. 30 min before test	Increased time in social compartment	[94]
mGluR2/3 antagonist	MGS0039	Sprague-Dawley rats	SIT	0.3, 1 and 3 mg/kg, i.p. 1 h before test	Did not affect social interaction	[95]
mGluR4 agonist	LSP4-2022	Wistar rats	SIT	0.5, 1 and 2 mg/kg, i.p. 45 min before test, 1 h 45 min after MK-801	Reduced MK-801-induced social interaction deficits	[102]
		Swiss Webster mice	SIT	0.1, 0.5 and 1 mg/kg, i.p. 45 min before MK-801, 10 min before test	1 mg/kg but not 0.1 and 0.5 mg/kg prevented MK-801-induced social interaction deficits	[105]
mGluR4 PAM	Lu AF21934	Wistar rats	SIT	0.2, 0.5 and 1 mg/kg, s.c. 60 min before test, 2.5 h after MK-801	0.5 mg/kg but not 0.2 and 1 mg/kg reversed MK-801-induced social interaction deficits	[103,104]
mGluR4 PAM	Lu AF32615	Wistar rats	SIT	2, 5 and 10 mg/kg, s.c. 60 min before test, 2.5 h after MK-801	10 mg/kg but not 2 and 5 mg/kg reversed MK-801-induced social interaction deficits	[103]
mGluR4 PAM	VU0155041	Oprm1-/- mice	SIT	5 mg/kg, i.p. for 8 days	Normalized social interaction deficits	[101]
mGluR7 NAM	ADX71743	Swiss mice	SIT	1, 5 and 15 mg/kg, i.p. 30 min before MK-801, 30 min before test	5 and 15 mg/kg but not 1 mg/kg prevented MK-801-induced social interaction deficits	[109]
mGluR7 NAM	MMPIP	Swiss mice	SIT	5, 10 and 15 mg/kg, i.p. 30 min before MK-801, 30 min before test	Did not prevent MK-801-induced social interaction deficits	[109]
		Sprague-Dawley rats	SIT	3, 10 and 30 mg/kg, i.p. 30 min before test	30 mg/kg but not 3 and 10 mg/kg decreased social interaction	[112]
mGluR8 PAM	AZ12216052	C57BL/6J mice	SIT	10 mg/kg, i.p. 2 h before test	Did not affect social interaction	[106]

mGluR, metabotropic glutamate receptor; PAM, positive allosteric modulator; NAM, negative allosteric modulator; JNJ16259685, (3,4-dihydro-2H-pyrano[2,3-b]quinolin-7-yl)-(cis-4-methoxycyclohexyl)-methanone; CFMTI, 2-cyclopropyl-5-[1-(2-fluoro-3-pyridinyl)-5-methyl-1H-1,2,3-triazol-4-yl]-2,3-dihydro-1H-isoindol-1-one; fenobam, 3-(3-chlorophenyl)-1-(1-methyl-4-oxo-5H-imidazol-2-yl)urea; MPEP, 2-methyl-6-phenylethynyl-pyridine; MTEP, 3-((Methyl-1,3-thiazol-4-yl)ethynyl) pyridine hydrochloride; CDPPB, [3-cyano-*N*-(1,3-diphenyl-1H-pyrazol-5-yl) benzamide]; GRN-529; ((4-difluoromethoxy-3-(pyridine-2-ylethynyl)phenyl)5H-pyrrolo[3,4-b]pyridine-6(7H)-yl methanone); LY354740, (1S,2S,5R,6S)-2-Aminobicyclo[3.1.0]hexane-2,6-dicarboxylic acid; LY379268, (–)-2-Oxa-4-aminobicyclo[3.1.0]hexane-4,6-dicarboxylic acid; LY341495, [(2S)-2-amino-2-[(1S,2S)-2-carboxycycloprop-1-yl]-3-(xanth-9-yl) propanoic acid]; MGS0039, (1R,2R,3R,5R,6R)-2-Amino-3-(3,4-dichlorobenzyloxy)-6-fluorobicyclo[3.1.0]hexane-2,6-dicarboxylic acid; LSP4-2022, (2S)-2-amino-4-({[4-(carboxymethoxy)phenyl](hydroxy)methyl}(hydroxy)phosphoryl)butanoic acid; Lu AF21934, (1S,2R)-N1-(3,4-dichlorophenyl)cyclohexane-1,2-dicarboxamide; Lu AF32615, (E)-4-(2-Phenylethenyl)-2-pyrimidinamine; VU0155041, *cis*-2-[[(3,5-Dichlorophenyl)amino]carbonyl]cyclohexanecarboxylic acid sodium salt; ADX71743, [6-(2,4-Dimethylphenyl)-2-ethyl-6,7-dihydro-4(5*H*)-benzoxazolone]; MMPIP, [6-(4-Methoxyphenyl)-5-methyl-3-(4-pyridinyl)-isoxazolo(4,5-c)pyridin-4(5H)-one; AZ12216052, 2-[[(4-Bromophenyl)methyl]thio]-*N*-[4-(1-methylpropyl)phenyl]acetamide; three-chambered SAT, three-chambered social approach test; SIT, social interaction test; s.c., subcutaneous; i.p., intraperitoneal; p.o., orally; MK-801, 5R,10S-(+)-5-methyl-10,11-dihydro-5H-dibenzo[a,d]cyclohepten-5,10-imine hydrogen maleate.

**Table 2:** Effects of pharmacological modulation of mGluRs on social memory.

Action	Drug	Species	Behavioral Test	Dose Used	Behavioral Change	References
mGluR1 antagonist	JNJ16259685	Sprague-Dawley rats	SRT with one stimulus, 30 min IEI	0.3 and 1 mg/kg, i.p. 90 min before MK-801, 60 min before test	Reversed MK-801-induced social memory impairment	[119]
mGluR5 PAM	ADX47273	Wistar rats	SD, 30 min IEI	0.16, 0.63, 2.5 and 10 mg/kg, i.p. 30 min before test	2.5 mg/kg but not 0.16, 0.63 and 10 mg/kg enhanced social discrimination	[121]
				0.16, 0.63 and 2.5 mg/kg/day, i.p. postnatal days 35–46	2.5 mg/kg but not 0.16 and 0.63 mg/kg reversed social memory impairment in neonatally PCP-treated adult rats	
mGluR5 PAM	CDPPB	Wistar rats	SD, 30 min IEI	0.16, 2.5, 10 and 40 mg/kg, i.p. 30 min before test	2.5 and 10 mg/kg but not 0.16 and 40 mg/kg enhanced social discrimination	[121]
				10 mg/kg, i.p. 15 min before MK-801, 45 min before test	Attenuated MK-801-induced social memory impairment	
				0.63, 2.5 and 10 mg/kg/day, i.p. postnatal days 35–46	10 mg/kg but not 0.63 and 2.5 mg/kg reversed social memory impairment in neonatally PCP-treated adult rats	
mGluR2 PAM	BINA	Sprague-Dawley rats	SRT with one stimulus, 30 min IEI	10 and 30 mg/kg, i.p. 90 min before MK-801, 60 min before test	30 mg/kg but not 10 mg/kg reversed MK-801-induced social memory impairment; effect blocked by pretreatment with the mGluR2/3 antagonist LY341495 (3 mg/kg, i.p.)	[119]
mGluR2 PAM	LY487379	Wistar rats	SD—30 min with one stimulus, last 5 min with two stimuli	3, 10 and 30 mg/kg, i.p. 30 min before test	10 and 30 mg/kg increased preference for social novelty in neonatally PCP-treated adult rats	[122]
mGluR2 PAM	TASP0443294	Sprague-Dawley rats	SRT with 1 stimulus, 30 min IEI	10 and 30 mg/kg, p.o. 2 h before MK-801, 30 min before test	30 mg/kg but not 10 mg/kg prevented MK-801-induced social memory impairment	[125]
mGluR2/3 agonist	LY354740	Wistar rats	SD—30 min with one stimulus, last 5 min with two stimuli	1, 3 and 10 mg/kg, i.p. 30 min before test	Increased preference for social novelty in neonatally PCP-treated adult rats	[122]
mGluR2/3 agonist	LY379268	Sprague-Dawley rats	SRT with one stimulus, 30 min IEI	0.3 and 1 mg/kg, s.c. 90 min before MK-801, 60 min before test	1 mg/kg but not 0.3 mg/kg reversed MK-801-induced social memory impairment	[119]
mGluR2/3 antagonist	MGS0039	Sprague-Dawley rats	SRT with one stimulus, 30 min IEI	0.3, 1 and 3 mg/kg, i.p. immediately after learning session	1 and 3 mg/kg but not 0.3 mg/kg enhanced social recognition	[118]

mGluR, metabotropic glutamate receptor; PAM, positive allosteric modulator; JNJ16259685, (3,4-dihydro-2H-pyrano[2,3-b]quinolin-7-yl)-(cis-4-methoxycyclohexyl)-methanone; BINA, 3′-[[(2-cyclopentyl-2,3-dihydro-6,7-dimethyl-1-oxo-1H-inden-5-yl)oxy]methyl]-[1,1′-biphenyl]-4-carboxylic acid; LY487379, 2,2,2-Trifluoro-*N*-[4-(2-methoxyphenoxy)phenyl]-*N*-(3-pyridinylmethyl)ethanesulfonamide hydrochloride; TASP0443294, (2S)-5-methyl-2-{[4-(1,1,1-trifluoro-2-methylpropan-2-yl)phenoxy]methyl}-2,3-dihydroimidazo[2,1-b][1,3]oxazole-6-carboxamide; LY354740, (1S,2S,5R,6S)-2-Aminobicyclo[3.1.0]hexane-2,6-dicarboxylic acid; LY379268, (–)-2-Oxa-4-aminobicyclo[3.1.0]hexane-4,6-dicarboxylic acid; MGS0039, (1R,2R,3R,5R,6R)-2-Amino-3-(3,4-dichlorobenzyloxy)-6-fluorobicyclo[3.1.0]hexane-2,6-dicarboxylic acid; ADX47273, (*S*)-(4-fluorophenyl)-3-[3-(4-fluoro-phenyl)-[1,2,4]-oxidiazol-5-yl]piperidon-1-yl)methanone; CDPPB, [3-cyano-*N*-(1,3-diphenyl-1H-pyrazol-5-yl) benzamide]; SRT, social recognition test; IEI, inter-exposure interval; SD, social discrimination test; i.p., intraperitoneal; MK-801, 5R,10S-(+)-5-methyl-10,11-dihydro-5H-dibenzo[a,d]cyclohepten-5,10-imine hydrogen maleate; p.o., orally; s.c., subcutaneous; LY341495, [(2S)-2-amino-2-[(1S,2S)-2-carboxycycloprop-1-yl]-3-(xanth-9-yl) propanoic acid]; PCP, phencyclidine.

**Table 3:** Effects of pharmacological modulation of mGluRs on male aggressive behavior.

Action	Drug	Species	Behavioral Test	Dose Used	Behavioral Change	References
mGluR1 antagonist	JNJ16259685	OF.1 mice	Standard opponent test	0.125, 0.25, 0.5, 1, 2, 4 and 8 mg/kg, i.p. 30 min before test	All doses reduced isolation-induced aggressive behavior (threat and attack)	[131]
mGluR5 antagonist	MPEP	OF.1 mice	Standard opponent test	5, 10, 15, 20 and 25 mg/kg, i.p. 30 min before test	Increased social investigation and decreased aggression (threat and attack)	[127]
mGluR5 antagonist	MTEP	Swiss Webster mice	Resident-intruder test	1, 3 and 5.6 mg/kg, i.p. 10–30 min before test	Reduced attack bite frequency	[129]
mGluR2/3 agonist	LY379268	Swiss Webster mice	Resident-intruder test	0.3, 1 and 3 mg/kg, i.p. 10–30 min before test	3 mg/kg but not 0.3 and 1 mg/kg reduced attack bite frequency	[129]
		ddY mice	Resident-intruder test	1 mg/kg, i.p. 60 min before test	Reduced isolation-induced aggressive behavior	[128]
mGluR7 agonist	AMN082	OF.1 mice	Standard opponent test	0.5, 1, 2 and 4 mg/kg i.p. 60 min before test	4 mg/kg decreased aggression (threat and attack) but did not affect social investigation	[132]
mGluR7 antagonist	MMPIP	C57BL/6N mice	Resident-intruder test	0.25 μL (5 μg/μL) in BNST	Decreased inter-male aggression	[130]
mGluR8 agonist	(S)-3,4-DCPG	OF.1 mice	Standard opponent test	2.5, 5 and 10 mg/kg i.p. 30 min before test	Did not affect aggressive behavior or social investigation	[132]
mGluR8 agonist and AMPAR antagonist	(RS)-3,4-DCPG	OF.1 mice	Standard opponent test	5, 10 and 20 mg/kg, i.p. 30 min before test	Did not affect aggressive behavior or social investigation	[133]

mGluR, metabotropic glutamate receptor; AMPAR, α-amino-3-hydroxy-5-methyl-4-isoxazolepropionic acid receptor; JNJ16259685, (3,4-dihydro-2H-pyrano[2,3-b]quinolin-7-yl)-(cis-4-methoxycyclohexyl)-methanone; MPEP; 2-methyl-6-phenylethynyl-pyridine; MTEP, 3-((Methyl-1,3-thiazol-4-yl)ethynyl) pyridine hydrochloride; LY379268, (–)-2-Oxa-4-aminobicyclo[3.1.0]hexane-4,6-dicarboxylic acid; AMN082, *N*,*N*′-*Bis*(diphenylmethyl)-1,2-ethanediamine dihydrochloride; MMPIP, [6-(4-Methoxyphenyl)-5-methyl-3-(4-pyridinyl)-isoxazolo(4,5-c)pyridin-4(5H)-one; (S)-3,4-DCPG, (*S*)-3,4-Dicarboxyphenylglycine; (RS)-3,4-DCPG, (*RS*)-3,4-Dicarboxyphenylglycine; i.p., intraperitoneal; BNST, bed nucleus of the stria terminalis.

**Table 4:** Effects of pharmacological modulation of mGluRs on male sexual behavior.

Action	Drug	Species	Dose Used	Behavioral Change	References
mGluR1 antagonist	LY367385	Japanese quail	100 μg, i.c.v, 30 min before test	Inhibited sexual motivation	[140]
mGluR5 antagonist	MPEP	Japanese quail	100 μg, i.c.v, 30 min before test	Did not affect sexual motivation	[140]
		Wistar rats	1 or 10 μg/μL, Nac, 15 min before test	Did not affect sexual behavior	[143]
		Sprague-Dawley rats	10 mg/kg, i.p. for 14 days	Did not affect sexual behavior (number of erections within 30 min)	[142]
		Long-Evans rats	10 and 20 mg/kg, i.p. 1 and 2 h before mating	Decreased sex-seeking behavior and inhibited sexual behavior at high doses	[139]
mGluR5 antagonist	MTEP	Wistar rats	1 μg/μL, Nac, 15 min before test	Did not affect sexual behavior	[143]
		Sprague-Dawley rats	10 mg/kg, i.p. for 14 days	Did not affect sexual behavior (number of erections within 30 min)	[142]
mGluR5 NAM	GRN-529	Sprague-Dawley rats	1 and 10 mg/kg, i.p. for 14 days	Did not affect sexual behavior (number of erections within 30 min)	[142]
mGluR2/3 agonist	LY379268	Long-Evans rats	1 and 3 mg/kg, i.p. 1 and 2 h before mating	Did not affect sex-seeking behavior	[139]
mGluR2/3 antagonist	LY341495	Japanese quail	100 μg, i.c.v, 30 min before test	Did not affect sexual motivation and sexual performance	[140]
mGluR7 agonist	AMN082	Long-Evans rats	3, 10 and 20 mg/kg, i.p. 1 and 2 h before mating	Decreased sex-seeking behavior and inhibited sexual behavior at high doses	[139]

mGluR, metabotropic glutamate receptor; NAM, negative allosteric modulator; LY367385, [(S)-(+)-α-amino-4-carboxy-2-methylbenzeneacetic acid]; MPEP; 2-methyl-6-phenylethynyl-pyridine; MTEP, 3-((Methyl-1,3-thiazol-4-yl)ethynyl) pyridine hydrochloride; GRN-529, ((4-difluoromethoxy-3-(pyridine-2-ylethynyl)phenyl)5H-pyrrolo[3,4-b]pyridine-6(7H)-yl methanone); LY379268, (–)-2-Oxa-4-aminobicyclo[3.1.0]hexane-4,6-dicarboxylic acid; LY341495, [(2S)-2-amino-2-[(1S,2S)-2-carboxycycloprop-1-yl]-3-(xanth-9-yl) propanoic acid]; AMN082, *N*,*N*′-*Bis*(diphenylmethyl)-1,2-ethanediamine dihydrochloride; i.c.v. intracerebroventricular; Nac, nucleus accumbens; i.p., intraperitoneal.
